# Peter Allebeck: a steward of science, a builder of communities, and a champion of European public health

**DOI:** 10.1093/eurpub/ckag042

**Published:** 2026-03-24

**Authors:** Martin McKee, Tiago Correia

**Affiliations:** Special Adviser, European Public Health Association, London, United Kingdom; Editor in Chief, European Journal of Public Health, Lisbon, Portugal

In 2010, Peter Allebeck became the fourth editor-in-chief of this journal. Peter’s editorial stewardship coincided with a period of remarkable growth and maturation for EJPH. Under his leadership, the journal has consolidated its position as a standard-bearer for rigorous, policy-relevant public health research, elevating debates on everything from social determinants to health system performance.

Journals are often judged in terms of bibliometric scores, and if that were all, then Peter’s tenure would be seen as a great success. But that is far too narrow an assessment of his leadership of a journal that, through its close links to EUPHA, is the voice of Europe’s public health community. Peter insisted on not only methodological rigour, clarity of purpose, and fair review. He also encouraged authors to engage with the practical implications of their findings for European societies. His stewardship helped the journal become a home for scholarship that is both exacting and outward-looking, attentive to the diversity of contexts across our region.

Beyond his editor’s desk, Peter’s own scientific record speaks to a life’s work at the interface of evidence and action. Based at the Karolinska Institute since 2006 and, before that, the University of Gothenburg, he has generated an extremely influential body of research across epidemiology, addiction, and health inequalities, with particularly notable contributions to our understanding of the health effects of alcohol and cannabis and the epidemiology of schizophrenia and suicide. Once again, the bibliometric measures tell a story of great success, but that is only part of the picture. He has made many extremely important contributions to policy debates on topics ranging from substance use and alcohol harms to population-level assessments of disease burden and life expectancy, often in collaboration with a wide network of European and global colleagues. Remarkably, he combined this prodigious research output and his editorial responsibilities with a five-year stint as Secretary General of the Swedish Research Council for Health, Working Life and Welfare. And, in 1998, while at Gothenburg, he was EUPHA president, hosting that year’s European Public Health Conference.

Those collaborations matter. Public health thrives when knowledge crosses borders and disciplines. Peter helped make that happen, both as an author and as an editor, by championing multicountry analyses and nurturing early-career researchers. Landmark collaborative efforts, such as his contributions to the Global Burden of Disease programme, have strengthened the evidence base that underpins prevention and policy across Europe. The journal’s growing importance as a vehicle for cross-national, policy-relevant work during his tenure is no coincidence. It is an expression of his conviction that public health scholarship should be both excellent and useful.

He is also an exemplar of the quiet virtues that sustain our scholarly commons: transparency, inclusivity, and collegiality. Some editors wait to see what is submitted to them. Not Peter. Early-career researchers across Europe have benefited from his gift for institution-building, as he motivates reviewers, mentors authors, and curates a broad, expert editorial community that speaks to Europe’s varied realities. These are the often invisible labours of editorship, yet they are decisive in shaping the quality and credibility of a journal.

Transitions warrant both gratitude and confidence. Gratitude for the culture of excellence and integrity Peter leaves behind. Confidence, because of the robust editorial architecture he helped to build and because of the journal’s smooth handover to new leadership.

It is also fitting to set Peter’s editorship in the broader trajectory of EJPH itself. Since its inception in 1991, the journal has served as a vital, pan-European forum, bridging researchers, practitioners, and policymakers. Under Peter’s stewardship, that forum has not only been preserved but also renewed: ever more open to interdisciplinary inquiry, attuned to equity, and committed to translating evidence into policy discourse.

What, then, is Peter’s legacy? It is, first, his research, producing a large and sustained contribution to understanding the determinants of health and harm in European populations. It is, second, his editorship, with 15 years of principled gatekeeping that improved papers, encouraged authors, increased the number of papers from previously under-represented countries, and enhanced the journal’s role in shaping policy-relevant science. Third, it is the networks of trust and collaboration he cultivated, across institutes, countries, and generations, that will continue to sustain European public health in the years to come. And fourth, and by no means least, even if it is likely the least visible, is his enormous contribution of wisdom and sound judgement to the work of EUPHA’s Executive Council.

On behalf of colleagues across Europe and beyond, we say thank you. Thank you for setting a high bar and helping us meet it. Thank you for insisting that excellence and usefulness are not opposing virtues but mutually reinforcing ones. And thank you for reminding us that a journal is more than its metrics; it is a living commons, sustained by integrity, curiosity, and care. As EJPH moves forward under new leadership, it will do so in a landscape you helped to shape. One in which rigorous, policy-relevant scholarship can flourish for the benefit of public health. The record of the journal, the public recognition of your service, and the enduring strength of the community you nurtured are testimony enough.

Note: MM was the second Editor-in-Chief of the *European Journal of Public Health* and is a past president of EUPHA.

